# Numerical study of instability of nanofluids: the coagulation effect and sedimentation effect

**DOI:** 10.1186/1556-276X-6-183

**Published:** 2011-02-28

**Authors:** Yu Ni, JianRen Fan, YaCai Hu

**Affiliations:** 1 State Key Laboratory of Clean Energy Utilization, Zhejiang University, Hangzhou 310027, P. R. China

## Abstract

This study is a numerical study on the coagulation as well as the sedimentation effect of nanofluids using the Brownian dynamics method. Three cases are simulated, focusing on the effects of the sizes, volume fraction, and ζ potentials of nano-particles on the formation of coagulation and sedimentation of nanofluids. The rms fluctuation of the particle number concentration, as well as the flatness factor of it, is employed to study the formation and variation of the coagulation process. The results indicate a superposition of coagulation and sedimentation effect of small nano-particles. Moreover, it is stable of nanofluids with the volume fraction of particles below the limit of "resolution" of the fluids. In addition, the effect of ζ potentials is against the formation of coagulation and positive to the stability of nanofluids.

## Introduction

The nanofluid is characterized by the fluid with nanometer-sized solid particles dispersed in solution [1], which can increase the heat transfer coefficient [2-6], enhance the critical heat flux in boiling heat transfer [7-9], reduce the wall friction force [10], improve the optical characteristics [11], etc. Nano-sized particles are utilized because of its better stability than the suspension of micro-sized particles. For a badly stable suspension, sedimentation or coagulation (agglomeration) may occur. It compromises the above-mentioned advantages of the nano-suspension.

As is well known [12], the occurrences of coagulation and sedimentation are the two main factors for the instability of nanofluid. The phenomenon of coagulation is characterized by the formation of particle clusters, i.e., particles are in contact with each other and the cohesion takes place. Then, the clusters grow up. Many researchers investigated the coagulation effect of particles by the Brownian dynamics simulation, focusing on the formation of gelation [13], coagulation rates [14], particle network [15], etc. For example, Hütter [14] identified the characteristic coagulation time scales in colloidal suspensions, and measured their dependencies on the solid content and potential interaction parameters. He also deduced different cluster-cluster bonding mechanisms in the presence of an energy barrier, etc. Besides, the sedimentation always occurs after a big particle cluster is established, i.e., the particles within the cluster sediment flow downward because of the increased effect of the gravity of the cluster over the buoyancy force of it, and reduced the effect of Brownian motion to the big cluster. Many researches were devoted to the sedimentation [16-18] using the Brownian dynamics simulation too. For example, Soppe and Jannsen [17] studied the sediment formation of colloidal particle by a process of irreversible single-particle accretion. They used the algorithm of Ermak and McCammon, incorporating the inter-particle forces and hydrodynamic interaction on the two-particle level, and analyzed the effect of two-particle hydrodynamic interactions on the sediment structure, etc. They found that the process of sediment formation by colloidal particle is the result of a delicate balance of sediment field strength, DLVO interactions, and hydrodynamic interactions.

However, there is an important issue about which few researches have been concerned: the interaction between the coagulation and sedimentation for the instability of nanofluids. For example, the processing of coagulation causes the particle clusters to grow up, and then the large clusters are more prone to sedimentation than that of small clusters because of the intensive gravity effect. In other words, the coagulation effect is able to augment the sedimentation effect. Thus, this study is intended to carry out some research on this issue, exploring the complex interaction as well as the close relation between the coagulation and the sedimentation phenomena.

## Numerical model

### Governing equation

In this study, the Brownian dynamics technique is employed to investigate the motion of nanoparticles. The governing equation is the so-called Langevin equation, which is formulated as follows [19]:

(1) $$r_{i}=r_{i}^{0}+\sum_{j}\frac{D_{ij}^{0}F_{j}^{0}}{k_{\text{B}}T}\Delta t+R_{i}(\Delta t)$$

where the superscript 0 indicates that the variable is corresponding to the beginning of the time step Δ*t*; *r*_*i *_is the *i*th component of the position vector of particle; *D*_*ij *_is the element of the diffusion tensor indexed by (*i*, *j*); *F*_*j *_is the force experienced by the *j*th particle; *k*_B_ is the Boltzmann constant; and *T *denotes the temperature. The displacement *R*_*i*_(Δ*t*) is a random displacement of a Gaussian distribution with a zero expectation and a ($2D_{ij}^{0}\Delta t$) covariance ($\langle R_{i}(\Delta t)R_{j}(\Delta t)\rangle =2D_{ij}^{0}\Delta t$). In this study, the Rotne-Prager tensor [20] is utilized as approximations to the hydrodynamic interaction:

(2) $$\left\{ \begin{array}{l} D_{ij}=\frac{k_{\text{B}}T}{6\pi \eta a}\delta_{ij},\;i,j\text{ on the same particle}\\ D_{ij}=\frac{k_{\text{B}}T}{8\pi \eta r_{ij}}\left[\left(\bar{\bar{I}}+\frac{{\bar{r}}_{ij}{\bar{r}}_{ij}}{r_{ij}^{2}}\right)+\frac{2a^{2}}{r_{ij}^{2}}\left(\frac{1}{3}\bar{\bar{I}}-\frac{{\bar{r}}_{ij}{\bar{r}}_{ij}}{r_{ij}^{2}}\right)\right] \end{array}\right.$$

where *η *is the viscosity, *a *is the particle radius, *δ*_*ij *_is the Kronecker delta, ${\bar{r}}_{ij}$ is the vector from the center of particle *i *to the center of particle *j*, and $\bar{\bar{I}}$ is the unit tensor.

Moreover, three forces, i.e., the attractive Van der Walls force, *f*_v_, the repulsive electrostatic force by the electric double layer, *f*_e_, and the gravity force *f*_g_ are considered as the forces experienced by any particle, which are formulated as follows [12]:

(3) $$F_{j}=f_{\text{V}}+f_{\text{e}}+f_{\text{g}}$$

(4) $$f_{\text{V}}=-\frac{Ad^{2}}{3}\cdot \frac{r_{ij}^{4}-0.5d^{4}}{r_{ij}^{3}{\left(r_{ij}^{2}-d^{2}\right)}^{2}}$$

(5) $$f_{\text{e}}=2\pi \varepsilon \left(\kappa \cdot \frac{d}{2}\right)\zeta^{2}\cdot \frac{e^{-\kappa (r_{ij}-d)}}{1+e^{-\kappa (r_{ij}-d)}}$$

(6) $$f_{\text{g}}=\left(\frac{1}{6}\pi d^{3}\right)\left(\rho_{\text{f}}-\rho \right)g$$

where *A*, *d*, *ε*, *κ*, *ζ*, *ρ*_f_, *ρ*, and g are the Hamaker constant, the particle diameter, the electric permittivity of the fluid, the inverse of the double-layer thickness, the zeta potential of the suspension, the density of the fluid, the density of the particle, and the gravity acceleration, respectively.

It is noted in Equation (4) that the results for *r*_*ij *_- d = 0 is meaningless when the contact between the two particles occur, and they will adhere to each other or rebound back. Thus, we treat the condition with $\tau =\frac{\left(r_{ij}-d\right)}{d}<\tau_{0}$ as the situation when the two particles are separated, so that Equation (4) works. Otherwise, it results in coalescence between the two particles. Once the coalescence between colliding particles takes place, the clusters start growing up.

### Simulation conditions

In this study, three cases with different diameters of particles (Case 1), different volume fractions (Case 2), and different zeta potentials (Case 3) are simulated, respectively (Table 1). For these cases, the parameters of the material, as well as other relevant parameters, are illustrated in Table 2.

**Table 1:** Three cases with different diameters of particles, volume fractions, and zeta potentials

Case 1: under different diameters *d*
(a) *d*_0_ = 10 nm, *N*_*p *_= 1200, *ψ *= 0.153, ζ = 0.0 eV
(b) *d*_1_ = 25 nm, *N*_*p *_= 1200, *ψ *= 0.153, ζ = 0.0 eV
(c) *d*_2_ = 50 nm, *N*_*p *_= 1200, *ψ *= 0.153, ζ = 0.0 eV
Case 2: under different volume fractions *ψ*
(a) *d*_1_ = 25 nm, *N*_*p *_= 400, *ψ *= 0.051, ζ = 0.0 eV
(b) *d*_1_ = 25 nm, *N*_*p *_= 1200, *ψ *= 0.153, ζ = 0.0 eV
(c) *d*_1_ = 25 nm, *N*_*p *_= 2100, *ψ *= 0.268, ζ = 0.0 eV
(d) *d*_1_ = 25 nm, *N*_*p *_= 4200, *ψ *= 0.537, ζ = 0.0 eV
Case 3: under different zeta potentials ζ
(a) *d*_1_ = 25 nm, *N*_*p *_= 1200, *ψ *= 0.153, ζ = 0 eV
(b) *d*_1_ = 25 nm, *N*_*p *_= 1200, *ψ *= 0.153, ζ = 0.01 eV
(c) *d*_1_ = 25 nm, *N*_*p *_= 1200, *ψ *= 0.153, ζ = 0.025 eV
(d) *d*_1_ = 25 nm, *N*_*p *_= 1200, *ψ *= 0.153, ζ = 0.05 eV

**Table 2:** Parameters used in this simulation

Simulation domains (*L*_*x*_, *L*_*y*_, *L*_*z*_)	(4*d*, 4*d*, 256*d*)
Mesh sizes (*δ*_*x*_, *δ*_*y*_, *δ*_*z*_)	(4*d*, 4*d*, 4*d*)
Temperature *T *(°C)	25
Hamaker constant *A *(*J*)	2 × 10^-20^
Diameters of particle (*d*_0_, *d*_1_, *d*_2_), (*nm*)	(10, 25, 50)
Density of fluid *ρ*_f_ (kg/m^3^)	0.993 × 10^3^
Density of particle (*ρ*_p_) (kg/m^3^)	6.4 × 10^3^
Viscosity of fluid *η *(Pa s)	1.0 × 10^-3^
Simulation time step Δ*t *(ns)	10

In this simulation, the boundary conditions in the *x *and *y *directions (Table 2) in the horizontal plane are both periodic, whereas the top and bottom walls of the simulation domain in the *z*-direction are treated as adhesive walls to which the particles adhere immediately once they come into contact with them. It is reasonable to conclude thus, since the agglomerated particle clusters always adhere to the bottom walls or the top interfaces.

Initially, a random distribution is given to the particles. As time advances, the possible movements of particles are computed through solution of the governing equations.

## Simulation results

### Case 1: effect of particle sizes

**Figure 1:**
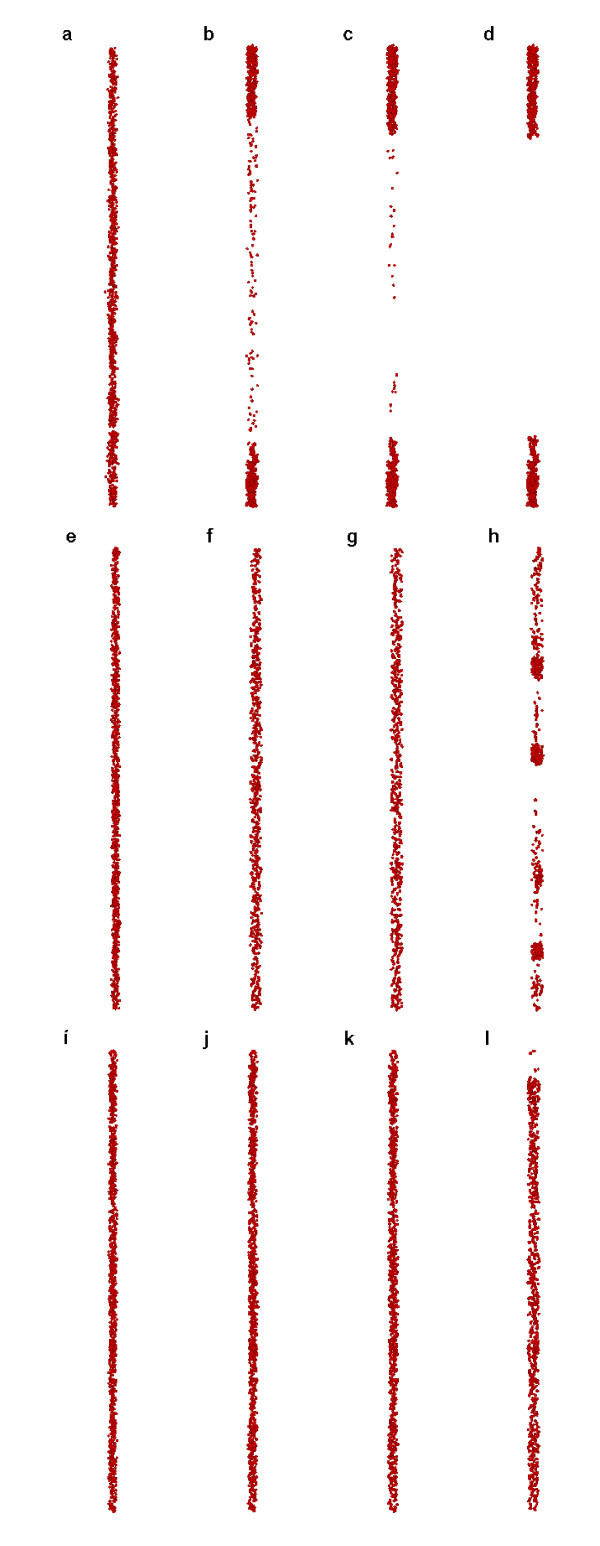
**Snapshots of simulation results**. (Case 1 for *d *= 10 nm **(a-d)**, *d *= 25 nm **(e-h)**, *d *= 50 nm **(i-l) **at *t *= 0, 5, 10, and 50 μs respectively).

This section deals with the effect of particle sizes on the coagulation and sedimentation. Figure 1 shows the simulation results at *t *= 0, 5, 10, and 50 μs for Case 1 due to the effects of different sizes of particles. Figure 1a, b, c, d shows the results of *d *= 10 nm at *t *= 0, 5, 10, and 50 μs, respectively. Similarly, Figure 1e, f, g, h shows the results for *d *= 25 nm, whereas Figure 1i, j, k, l shows them for *d *= 50 nm. It is seen that the coagulation takes place the most intensively and rapidly for the smallest size of particles (Figure 1a, b, c, d), moderately for the intermediate size of particles (Figure 1e, f, g, h), and weakly and slowly for the largest size of particles (Figure 1e, f, g, h, i, j, k, l). More importantly, the results of the intermediate sizes are due to coagulation but with weak sedimentation, whereas the results of the smallest sizes are due to both the effects of coagulation and sedimentation. For the largest size of the particles, it is neither due to coagulation nor sedimentation. It looks complicated. As is known, the larger particles bear the major effect of gravity, and they are the most prone to sedimentation. However, it is only true of the single particle without coagulation. With the superposition of the coagulation effect, it can amplify or augment the trend of sedimentation through coagulation. Owing to the increasing agglomeration of the particles, the gravity effect may play an important and even a dominant role, which causes possible sedimentation of the whole agglomeration (the upper part of the agglomeration in Figure 1d is due to the adhesive boundary on the upper wall). In other words, there exists a balance between the sedimentation effect of the large-sized individual particles and the sedimentation effect of small-sized aggregated particles. The former is caused solely by the gravity effect, whereas the latter is caused by the superposition of the coagulation and the gravity effects, i.e., the amplification and augmentation of the gravity effects of the aggregated particles due to coagulation.

It is necessary to mention that Figure 1i, j, k, l does not indicate the stability of the nanofluids. Alternatively, it indicates a relatively stable characteristic compared to Figure 1a, b, c, d. After the evolution over a long time, possible coagulation or sedimentation may also occur.

In order quantify the degree of coagulation, we need to define some functions. Let us divide the simulation domain *L*_*x *_× *L*_*y *_× *L*_*z *_by *N*_*x *_× *N*_*y *_× *N*_*z *_cubic meshes by the cell volume (*δ*_*x *_× *δ*_*y *_× *δ*_*z*_). The mean number concentration $\bar{c}$ of particles is the mean number of particles within each mesh volume (*δ*_*x *_× *δ*_*y *_× *δ*_*z*_). Then, the rms value of the particle concentration **R**_1_ and the flatness factor of the number concentration **R**_4_ are formulated as follows:

(7) $${\text{R}}_{\text{1}}={\left(\frac{1}{L_{z}}\int {\left(c(x,y,z)-\bar{c}\right)}^{2}dz\right)}^{1/2}$$

(8) $${\text{R}}_{4}={\left(\frac{1}{{\text{R}}_{\text{1}}}\right)}^{4}\frac{1}{L_{z}}\int {\left(c(x,y,z)-\bar{c}\right)}^{4}dz$$

The rms of concentration means the fluctuation of the number concentration of particles, and it is closely related to the formation of particle clusters due to coagulation. The flatness factor means the intensity of fluctuation of the number concentration, thereby indicating the intensity of coagulation. Thus, these two functions are helpful in enabling the quantification of particle coagulation.

**Figure 2:**
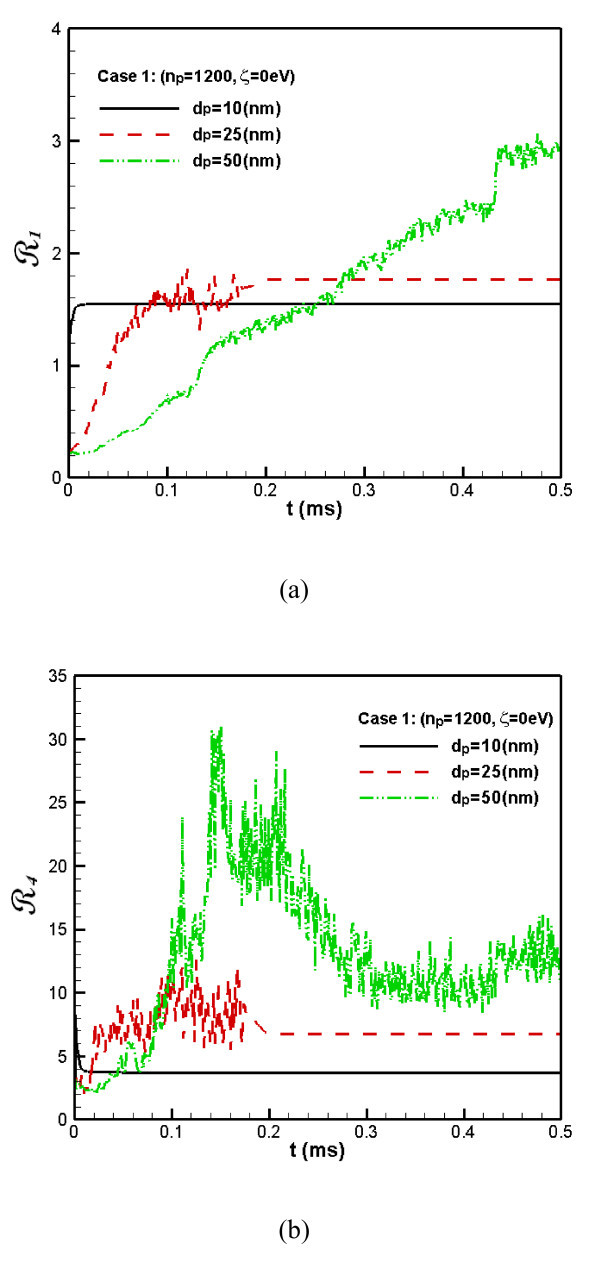
**The flatness factor of the concentration distribution of nanoparticles**. (The **R**_1_**(a) **and **R**_4_**(b) **for Case 1).

Figure 2a, b shows the **R**_1_ and **R**_4_ for Case 1. It is seen from Figure 2a, b that the coagulation of the small particles is the fastest. They become almost totally coagulated immediately even at the beginning. Comparatively, the coagulation of the larger particles takes place slowly and increasing steadily. However, the final level of coagulation of the larger particles is greater than that of smaller particles. In addition, the degree as well as the rapidity of the coagulation of the intermediate particles is intermediate between that of the smaller and the larger particles.

### Case 2: effects of volume fractions

In this section, the effect of volume fraction, i.e., the concentration of particles, is studied. As aforementioned, the smaller particles are more prone to coagulate than the larger particles, under the same condition of the volume fractions. However, the process of coagulation is also closely related to the number of particles contained in it.

**Figure 3:**
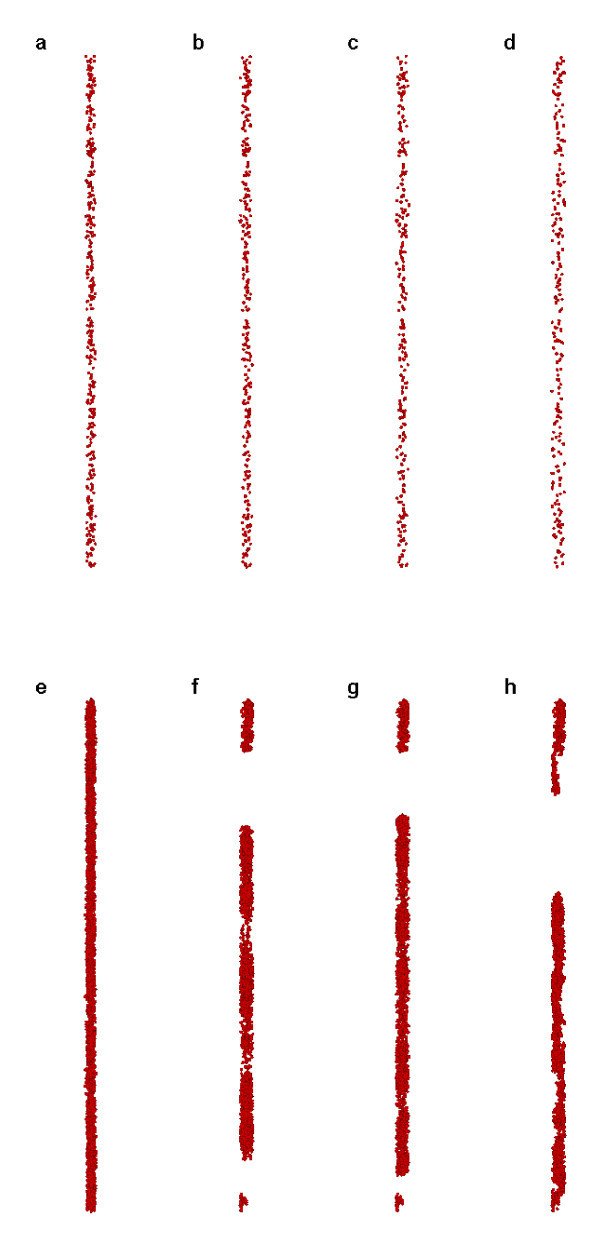
**Snapshots of simulation results**. (Case 2 for *n*_*p *_= 400 **(a-d) **and *n*_*p *_= 4200 **(e-h) **at *t *= 0, 5, 10, and 50 μs respectively).

For example, Figure 3a, b, c, d shows one of the results of Case 2 where only *n*_*p *_= 400 particles are simulated. Compared to Figure 1a, b, c, d, it is seen that the coagulation does not takes place at *t *= 50 μs. It says that the coagulation is to be regarded appropriately as a process with an excess of particle content. When the particle numbers go beyond the superior limit of the resolvent, then the coagulation will certainly take place. Thus, when the *n*_*p *_= 4200 particles are simulated, more intensive coagulations are observed correspondingly (Figure 3e, f, g, h).

**Figure 4:**
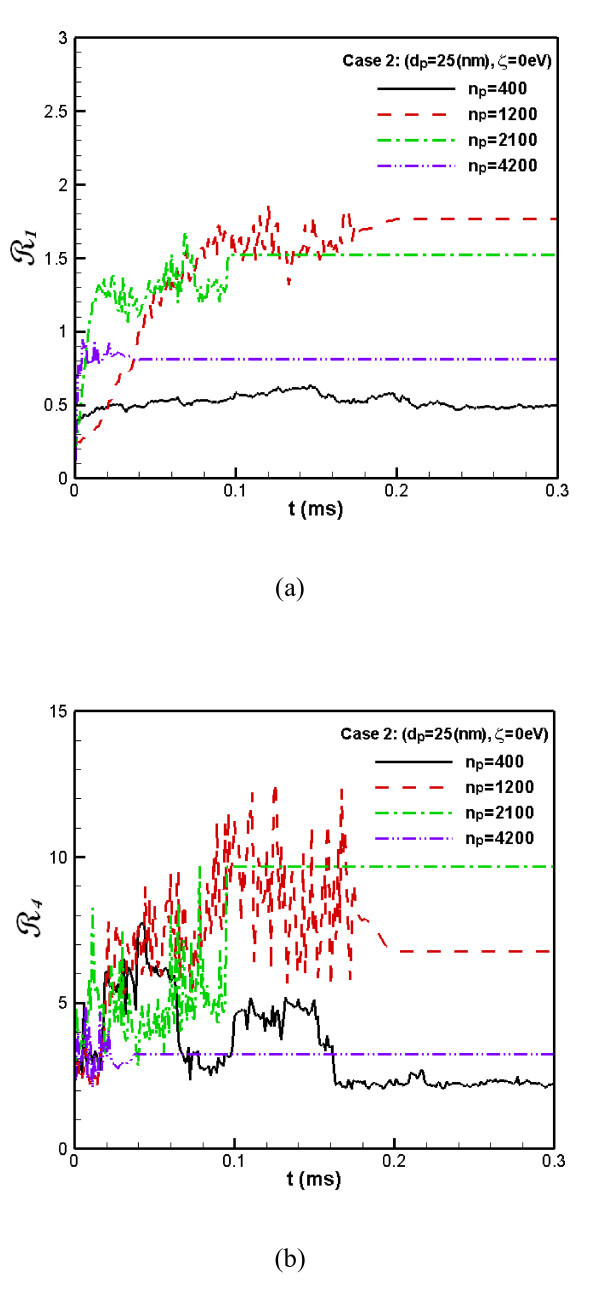
**The flatness factor of the concentration distribution of nanoparticles**. (The **R**_1_**(a) **and **R**_4_**(b) **for Case 2).

In addition, Figure 4a, b shows the **R**_1_ and **R**_4_ of Case 2. It is seen from Figure 4 that the **R**_1_ and **R**_4_ for *n*_*p *_= 400 are always relatively of small value, indicating a stable status almost without coagulation and sedimentation, although **R**_4_ is slightly fluctuated when *t *< 0.06. Moreover, compared to *n*_*p *_= 1200, it is seen that the concentration fluctuation **R**_1_ and flatness factor of concentration **R**_4_ for *n*_*p *_= 400 are relatively of lower values. It validates the conclusions derived from the observation of Figure 3a, b, c, d, in comparison with Figure 1e, f, g, h.

With the increased number of particles, it is seen that the **R**_1_ and **R**_4_ are increased too (*n*_*p *_= 1200 and 2100, respectively, Figure 4). However, when the particle number is extremely large, all the spaces are almost stuffed with particles, leading to a homogeneous distribution and a low fluctuation in the number concentration (*n*_*p *_= 4200, in Figure 4).

### Case 3: effects of ζ potentials

The previous sections showed the results with ζ = 0 eV. As seen from Equation (5), no repulsive effect has been considered between the particles since *f*_e_ = 0. Thus, this section will focus on the effect of the repulsive effect by varying the ζ potentials.

**Figure 5:**
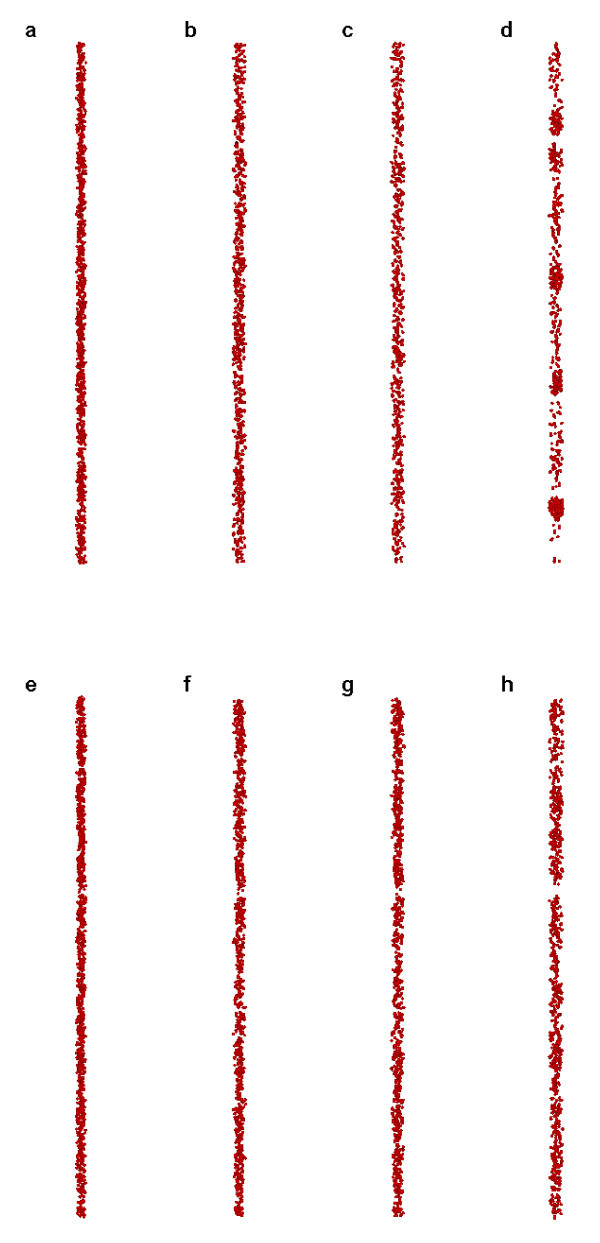
**Snapshots of simulation results**. (Case 3 for ζ = 0.01 eV **(a-d) **and ζ = 0.05 eV **(e-h) **at *t *= 0, 5, 10, and 50 μs, respectively).

Comparing with Figure 1e, f, g, h, it is seen that the degree of coagulation with ζ = 0.01 eV is attenuated (Figure 5a, b, c, d), and it almost disappears with ζ = 0.05 eV (Figure 5e, f, g, h). It indicates that the repulsive effect induced by the ζ potentials is beneficial to the stability of nanofluids, since it acts against the coagulation process.

**Figure 6:**
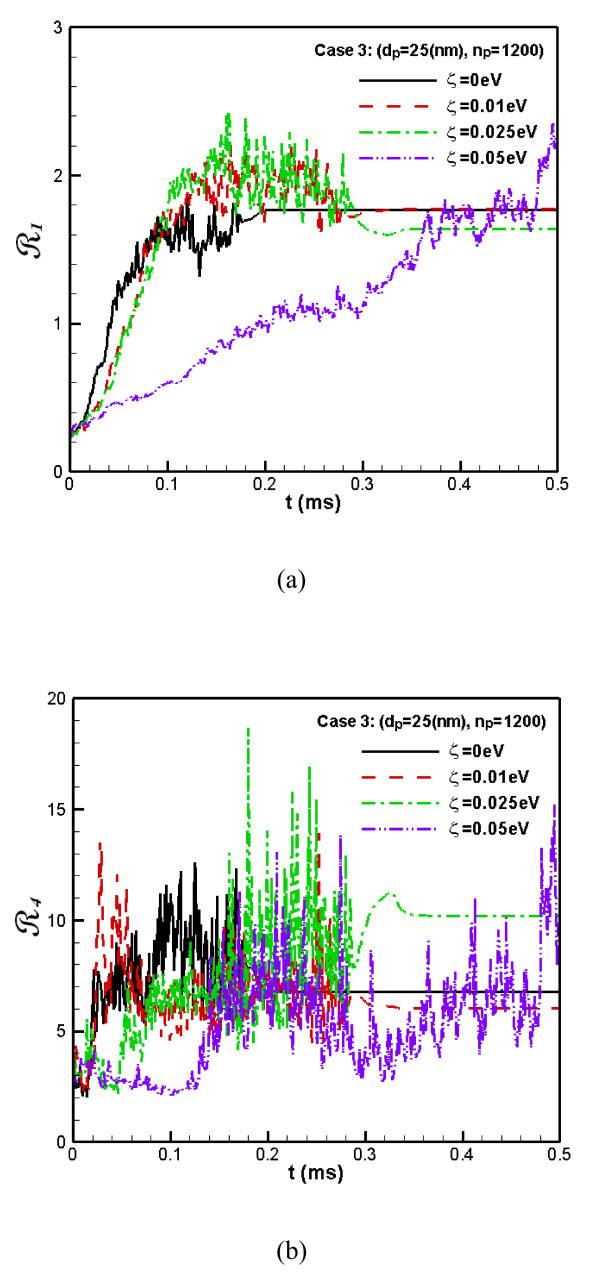
**The flatness factor of the concentration distribution of nanoparticles**. (The **R**_1_**(a) **and **R**_4_**(b) **for Case 3).

This conclusion is also validated by the variations of **R**_1_ and **R**_4_ (Figure 6a, b, respectively). It is seen that the fluctuation of the number concentration of particles with ζ = 0.05 eV increases much more slowly than the cases with smaller ζ. Although the **R**_1_ and **R**_4_ for ζ = 0.05 eV are still increasing with time, which indicates that the coagulation process may still take place after a fairly long time, their effects against the formation of coagulation are very clear. In other words, it is positively beneficial for the stability of nanofluids.

## Conclusions

The findings of this study are briefly summed up as follows:

1. A complicated superposition of the coagulation and sedimentation effects for small particle is observed. The mechanisms of sedimentation for the larger and the smaller particles are different. The former is caused mainly by the great gravity effect of any individual particle, whereas the latter is mainly due to the coagulation process, and the superposition of coagulation causes the sedimentation of the whole agglomeration of particles.

2. There exists a superior limit of the fluid for particle content. When the volume fraction is below the limit, it is hard for the coagulation to occur. In contrast, the coagulation will certainly take place when the concentration of nanoparticles is beyond the capacity of "resolution" of the fluids.

3. The effect of ζ potentials is beneficial for the stability of nanofluid, since it resists the formation of coagulation. In other words, increase in the value of ζ potentials is helpful to make the nanofluid more stable.

## Competing interests

The authors declare that they have no competing interests.

## Authors' contributions

All authors contributed equally.
